# Hepatitis E Virus Serosurvey among Pet Dogs and Cats in Several Developed Cities in China

**DOI:** 10.1371/journal.pone.0098068

**Published:** 2014-06-04

**Authors:** Huanbin Liang, Jidang Chen, Jiexiong Xie, Long Sun, Fangxiao Ji, Shuyi He, Yun Zheng, Chumin Liang, Guihong Zhang, Shuo Su, Shoujun Li

**Affiliations:** MOA Key Laboratory of Animal Vaccine Development, College of Veterinary Medicine, South China Agricultural University, Guangzhou, China; Virginia Polytechnic Institute and State University, United States of America

## Abstract

Infection by Hepatitis E virus (HEV), as a zoonotic disease virus, is well studied in pigs in China, but few studies in pets have been performed. This study was designed to characterize the prevalence of HEV infection among pet dogs and cats in major metropolitan areas of China. We conducted a seroepidemiological survey from 2012 to 2013 in 5 developed cities, Beijing, Shanghai, Canton, Shenzhen and Macao, by enzyme-linked immunosorbent assay (ELISA). The overall HEV seroprevalence in 658 dog and 191 cat serum samples was 21.12% and 6.28%, respectively. The analysis in dogs suggested that there were significant differences among cities, and the positive rate of HEV-specific antibody in all cities ranged from 6.06% (Shenzhen) to 29.34% (Beijing). Older pet cats have a high risk (OR, 10.25) for HEV seropositivity, but no strong relationship was observed between different genders and age groups. Additionally, it was revealed that stray dogs, omnivorous pet dogs and pet cats who share food, such as kitchen residue, with the general population would have a higher risk for HEV seropositivity. The odds ratios for these groups are 2.40, 2.83 and 5.39, respectively, compared with pet dogs and cats fed on commercial food. In this study, we first report that HEV is prevalent in pet dogs and cats in several large cities in China. Swill and kitchen residue may be a potential risk for HEV transmission from human to pets. As the sample size was relatively small in this study and may not be fully representative of China, further investigation is required to confirm the conclusions.

## Introduction

Hepatitis E virus (HEV) is a self-limiting small non-enveloped RNA virus of the genus *Hepevirus* in the family *Hepeviridae* [1]. There are 4 HEV genotypes but only 1 serotype. Genotypes 1 and 2 infect only humans, but genotypes 3 and 4 infect humans, pigs and other animal species in America, Europe and Asia. The genotypes clearly differ with respect to host species [2], [3]. A wide variety of animals have been found to be reservoirs or sources of HEV infection [4]–[7]. With growing urbanization, more and more people keep pets, especially in economically developed areas. An epidemiological study was performed to find out if pet dogs and cats play an important role in the transmission of HEV in Japan and the result actually demonstrated the present of HEV-seropositive in cat [8]. There was a recent study regarding the sporadic acute hepatitis E of a 47-year-old man whose pet cat was positive for the antibody to hepatitis E virus [9]. In the Jiang-Zhe area of China, a survey demonstrated that the seroprevalence of HEV in pet dogs was approximately 13.5%, and positive serum from dogs could be reacted against swine HEV antigen [10]. For these reasons, pet dogs and cats caught our attention as a potential source of HEV transmission. However, there were few reports on the prevalence status of HEV among these pet animals in China. In this study, we investigated the prevalence of anti-HEV antibodies in the sera of pet dogs and cats from several large cities to produce a much more comprehensive serosurvey in China.

## Materials and Methods

### Serum samples, study area and categories for the animals

The blood samples from pet dogs and cats used for HEV serology were collected beforehand from different cities in China and stored at −70°C until tested. In total, 658 dog and 191 cat serum specimens were obtained from 37 different animal hospitals distributed in the most developed districts of the Beijing, Shanghai, Canton, Shenzhen and Macao districts from 2012 to 2013. In addition, 62 serum samples from stray dogs were collected from shelters located in Canton. Serum was collected before vaccination from healthy pets and stray dogs with local veterinarians' diagnosis and help. The animals (dogs and cats) were divided into different categories based on food sources. There are three distinct groups for dogs (stray dogs, omnivorous pet dogs, and pet dogs that fed on commercial dog food) and two separate groups for cats (omnivorous pet cats, and pet cats that fed on commercial cat food).

### Ethical Considerations

All the owners of the dogs and cats gave permission for their animals' sera to be used in this study. Our sampling processes were assisted by local authorities and veterinarians. Serum sample collection method was conducted under the guidance of the South China Agricultural University Experimental Animal Welfare Ethics Committee. The serosurvey in our study had been approved by the animal welfare ethics committee and the contract-numbers of the approval documents is 2013–04.

### Detection of antibodies against HEV in serum

To detect the total antibodies against HEV (anti-HEV), a commercial ELISA kit from Wantai Biopharmaceutical, Inc. (Beijing, China) was used. The laboratory analysis was performed according to the manufacturer's instructions [11]–[13]. This commercial kit was a double-antigen sandwich ELISA (DS-ELISA) kit. This kit is using the recombinant HEV ORF2 (amino acids 394 to 604) named E2 protein as antigen. It is demonstrated as a species independent assay detecting HEV IgG, IgM, and IgA and has been reported to have an overall specificity of 98.8% for human samples [14]–[16]. Also the dot-blot analysis of dog sera against swine HEV antigen showed that it was advisable to use this method for testing [10].

### Statistical analysis

The Fisher exact test and chi-square test were used to analyze the categorical variables when appropriate. The magnitude of the association between the variables and seropositivity is expressed as an odds ratio (OR) with 95% confidence intervals (95% CI). Univariate analysis was performed to identify which variables were significantly associated with anti-HEV seropositivity. We performed the statistical analysis using SPASS and GraphPad Prism (version 5.1). Statistical significance was set at p<0.05.

## Results

### Seroprevalence of HEV in dogs

The dog sera were divided into 5 groups based on the cities they were collected from. Among the 658 dog serum samples collected from Beijing, Canton, Macao, Shanghai and Shenzhen, the overall seroprevalence was 21.12% (139/658). The positive rate of HEV-specific antibody in all groups ranged from 6.06% to 29.34% (TABLE 1). Beijing had an approximately 6.5-fold higher risk than Shenzhen (odds ratio [OR], 6.44; 95% confidence interval [CI], 2.69–15.38; p<0.01). Macao (OR, 2.58; 95% CI, 0.87–7.66; p<0.01), Canton (OR, 3.56; 95% CI, 1.40–9.04; p<0.01), and Shanghai (OR, 4.72; 95%CI, 1.87–11.93; p<0.01) also had a higher risk.

**Table 1:** Prevalence of Anti-HEV in dog serum samples collected from five developed cities in China.

City	No. of serum samples	No. of positive serum samples	Analysis		
			positive rate(%)	OR(95%CI)	P value
Shenzhen	99	6	6.06	ref	
Macao	63	9	14.29	2.58(0.87–7.66)	>0.05
Canton	134	25	18.66	3.56(1.40–9.04)	<0.01*
Shanghai	120	28	23.33	4.72(1.87–11.93)	<0.01*
Beijing	242	71	29.34	6.44(2.69–15.38)	<0.01*

Six hundred and fifty-eight dog serum samples were collected from 37 pet hospitals since 2012. OR, odds ratio; CI, confidence interval; ref, reference; *, statistically significant.

Univariate analysis was used to compare the HEV seroprevalence rates among cities. There was no significant difference among the age groups (TABLE 2). Male dogs had an approximately 1.8-fold (OR, 1.84; 95% CI, 1.24–2.72; p<0.01) higher risk than female dogs (Fig. 1.1), and omnivorous dogs had an approximately 2.83-fold (OR, 2.83; 95% CI, 1.43–5.6; p<0.01) higher risk than dogs fed on dog food (Fig. 1.2).

**Figure 1:**
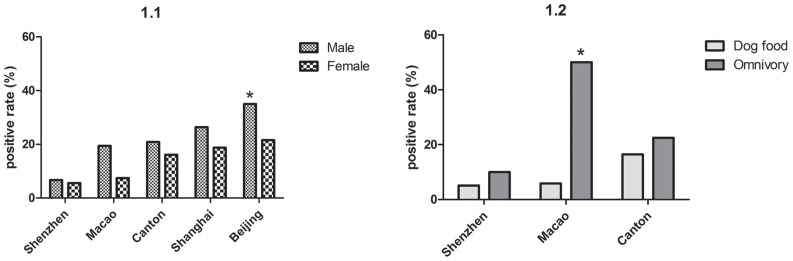
Prevalence of HEV among pet dogs in different cities according to gender and food. 1.1 Anti-HEV of dog serum for different genders; 1.2 Anti-HEV of dog serum for different eating habits. Statistical analyses performed in Fig. 1.1 and 1.2 separately divide by gender and food source for each city were indicated on the figure with an asterisk (*); *, statistically significant and P value <0.01.

**Table 2:** Anti-HEV in pet dogs in several developed cities of China according to different age groups, genders and food sources.

	Developed cities of China					Total			
	Shenzhen	Macao	Canton	Shanghai	Beijing				
	Positive rate%(Positive/Total)					% (positive/total)	OR (95% CI)	X^2^	P value
Age(year)									
≤1	0.00%(0/39)	0.00%(0/3)	30.43%(7/23)	15.00%(3/20)	30.23%(26/86)	21.05%(36/171)	ref		
2–5	8.33%(3/36)	0.00%(0/15)	17.50%(7/40)	21.43%(9/42)	34.88%(30/86)	22.37%(49/219)	1.08(0.66–1.76)	0.10	>0.05
6–10	5.00%(1/20)	7.70%(2/26)	20.45%(9/44)	26.32%(10/38)	22.92%(11/48)	18.75%(33/176)	0.87(0.51–1.47)	0.29	>0.05
≥10	50.00%(2/4)	36.84%(7/19)	7.41%(2/27)	30.00%(6/20)	18.18%(4/22)	22.83%(21/92)	1.11(0.60–2.04)	0.11	>0.05
Gender									
Female	5.56%(3/54)	7.41%(2/27)	16.13%(10/62)	18.75%(9/48)	21.57%(22/102)	15.70%(46/293)	ref		
Male	6.67%(3/45)	19.44%(7/36)	20.83%(15/72)	26.39%(19/72)	35.00%(49/140)	25.48%(93/365)	1.84(1.24–2.72)	9.33	<0.01*
Food									
Dog food	5.06%(4/79)	5.89%(3/51)	16.47%(14/85)	NA	NA	9.77(21/215)	ref		
Omnivory	10.00%(2/20)	50.00%(6/12)	22.45%(11/49)	NA	NA	23.46(19/81)	2.83(1.43–5.61)	9.43	<0.01*

OR, odds ratio; CI, confidence interval; *, statistically significant; X^2^, Chi-Square Test; ref, reference; NA, not analyzed; Dog food, commercially available and not fresh meat.

The survey tested the pet dog serum samples from 9 different districts of the urban and inner suburban areas of Beijing (TABLE 3). The positive rates of pet dog HEV-antibody in all areas ranged from 17.65% to 36.84%. However, no statistically significant difference in HEV seropositivity was found in different groups. In Canton, dog serum samples were separately collected from animal hospitals and stray dog shelters to determine whether being a stray was a risk factor. The results indicated that stray dogs had an approximately 2.4-fold (OR, 2.40; 95% CI, 1.45–4.18; p<0.05) higher risk than pet dogs, and the difference was statistically significant (TABLE 3).

**Table 3:** Seroprevalence of dog HEV infection in Beijing and Canton.

	Subject		Univariate analysis		
	Positive/Total(n)	Positive rate(%)	OR(95% CI)	X^2^	P value
Urban and inner suburban area of Beijing					
Beijing city	6/21	28.57	ref		
Chaoyang	18/57	31.58	1.15(0.38–3.47)	0.07	>0.05
Chongwen	4/17	23.53	0.77(0.18–3.34)	NA	>0.05
Dongcheng	7/19	36.84	1.46(0.39–5.51)	0.31	>0.05
Fengtai	7/21	33.33	1.25(0.34–4.64)	0.11	>0.05
Haidian	15/48	31.25	1.14(0.37–3.51)	0.05	>0.05
Shijingshan	5/19	26.32	0.89(0.22–3.60)	0.03	>0.05
Xicheng	3/17	17.65	0.54(0.11–2.57)	NA	>0.05
Xuanwu	6/23	26.09	0.88(0.23–3.33)	0.03	>0.05
Canton					
Pet dogs	25/134	20.20	ref		
Stray dogs	22/62	35.48	2.40(1.45–4.18)	6.58	<0.05*

OR, odds ratio; CI, confidence interval; *, statistically significant; X^2^, Chi-Square Test; NA, Fisher's exact test with no value; ref, reference.

### Detection of HEV-antibody in pet cats

As shown in TABLE 4, seroprevalence of pet cat serum samples collected from Canton and Shenzhen (2012–2013) was 6.28% (12/191). However, the positive rate in omnivore pet cats (17.65%, 6/34) was much higher than in cats fed on cat food (3.82%, 6/157). The OR value between them was 5.39 (95% CI, 1.62–17.93; p<0.01).

**Table 4:** Prevalence of antibody against HEV among pet cats by demographics.

	Subject		Univariate analysis		
	Positive/Total(n)	Positive rate(%)	OR(95% CI)	X^2^	P value
Age(year)					
≤1	1/42	2.38%	ref		
2–5	2/70	2.86%	1.21(0.11–13.73)	NA	≈1
6–10	2/44	4.55%	1.95(0.17–22.38)	NA	≈1
>10	7/35	20.00%	10.25(1.19–88.01)	NA	<0.05*
Gender					
Male	7/126	5.56%	ref		
Female	5/65	7.69%	1.42(0.43–4.65)	0.33	>0.05
Food					
Cat food	6/157	3.82%	ref		
Omnivory	6/34	17.65%	5.39(1.62–17.93)	9.07	<0.01**

One hundred and ninety-one pet cat serum samples were selected mainly from Canton and Shenzhen since 2012.

OR, odds ratio; CI, confidence interval; **, significantly different; *, different; X^2^, Chi-Square Test; NA, Fisher's exact test with no value; ref, reference; Cat food, commercially available and not fresh meat.

Gender seemed to have no relationship with pet cat HEV seroprevalence (OR, 1.42; 95%CI, 0.43–4.65; p>0.05). Samples at ages ≤1, 2–5, 6–10 and >10 years were examined (TABLE 4). As shown in TABLE 4, the different age groups show no significant association with HEV seropositivity. However, the trendline (data not shown) of pet cat anti-HEV showed a steeper slope in the >10 period (OR, 10.25; 95% CI, 1.19–88.01; 0.01<p,0.05).

## Discussion

According to the reports describing HEV seropositivity in dogs in India, Brazil and Eastern China, 22.7%, 17.8% and 6.97% of the dogs, respectively, were anti-HEV antibody-positive [17]–[19]. In the current study, the prevalence of anti-HEV in dog serum samples collected from five developed cities in China was analyzed. As shown in TABLE 1, the overall prevalence was 6.06%, 14.29%, 18.66%, 23.33% and 29.34% in Shenzhen, Macao, Canton, Shanghai and Beijing, respectively. The 95% confidence intervals are relatively wide, which might be caused by the small sample size. The large difference (based on odds ratios) between cities might prompt the possibility of geographical limitations. Compared with Shenzhen, the other cities seemed to have significant differences, and Beijing had the highest odds ratio in this study (OR, 6.44; 95% CI, 2.69–15.38). As shown in TABLE 3, the samples collected from the urban and inner suburban areas of Beijing included nine different districts (TABLE 3). The overall seroprevalence ranged from 17.65% to 36.84% in these areas, but these differences were not statistically significant. These characteristics of HEV prevalence in pet dogs might be caused by the geographical limitation. There was also no significant difference among age groups in the five different developed cities (TABLE 2). As reported in several surveys [13], [20]–[21], the seroprevalence of human or swine HEV IgG commonly increased with age. However, this study indicates the opposite conclusion: the positive rates for HEV antibody in pet dogs showed no significant relationship with age. To confirm this result, long term surveillance of HEV antibody levels in pets would be helpful.

In Korea, a survey indicated that the prevalence of anti HEV-reactive antibodies in cats was 8.1% [22]. Other earlier studies in Japan and China found anti-HEV positive cat serum samples [8], [23]. In this study, the seroprevalence of HEV in pet cat serum samples collected mainly from Canton and Shenzhen (2012–2013) was unexpectedly low (6.28%, 12/191), and most of the positive cats were aged. Only the >10 years age group showed a statistically high risk (approximately 10-fold) compared with other age groups (TABLE 4). This result suggested that seropositive of HEV was connected with pet cats age, but may not show a rigorous age dependence of the positivity rate for HEV antibodies. However, old pet cats had an obviously high positive rate (20.0%, 7/35) and might raise the risk of cat-to-human HEV transmission. In a previous study, an HEV patient in Japan had a pet cat that was reported to be HEV-positive [9]. Although that observation does not necessarily mean the virus transmission occurred from the cat to the human, the potential risk of cat-to-human transmission still exists. In order to understand the epidemiology of HEV infection in humans and other animals, a full-length HEV genome isolated from cats should be analysis.

As shown in Fig. 1.1, anti-HEV was generally higher in males than females in pet dogs in all five different cities (OR, 1.84; 95% CI, 1.24–2.72). However, in pet cats, no statistical difference was found (OR, 1.42; 95%CI, 0.43–4.65). The relationship between different genders of pets was weak enough that it might not represent a risk factor for HEV infection. Rodents are one probable reservoir, especially for companion animals, and it has recently been reported in Japan and China that wild rats have been found to be infected with HEV [24], [25]. A recent study suggested that rodent hunting by cats could be a reason for the higher levels of HEV antibodies [8]. Interestingly, we demonstrated that dogs have a higher positive rate than cats (21.12% vs. 6.28%). There may be some common infectious source affecting both pet dogs and pet cats because of the same living environment they share with human today. The different ELISA assays, different experimental conditions and the same living condition could be the reason of the discrepancy between the results of this study and others.

In Canton, both pet dogs and stray dogs were incorporated into this survey (TABLE 3). It was suggested that stray dogs have a higher risk of seropositivity than pet dogs (OR, 2.40; 95% CI, 1.45–4.18). Furthermore, in this study, pet dogs and cats were separated into two groups based on diet. Pet dogs from 3 different cities in the South of China (Canton, Shenzhen and Macao) presented similar results that omnivorous dogs had higher risk of HEV than those eating dog food in all three cities (Fig. 1.2). Nevertheless, between the three cities, dogs in Macao eating omnivorously actually appear to have a much higher risk of HEV seropositivity than those on dog food in comparison to Canton and Shenzhen. Similarly, omnivorous pet cats were conjectured to have a higher risk than pet cats that eat commercial food (OR, 5.39; 95% CI, 1.62–17.93). This results suggest that the dietary habits of pet dogs and cats should be considered one of the possible potential risk factors for HEV seropositivity and transmission. However, the commercial animal food which is rich in animal derived protein should be taken into consideration if it is a obstacle to this conjecture. As a previous report described, kitchen residue might play an important role in the human-to-pig transmission of HEV [26]. This study revealed that stray dogs and omnivorous pet dogs and cats that share food (including swill and kitchen residue) with the general population have a higher risk for HEV seroconvesion. Further investigation is required to determine the prevalence of HEV in dogs and cats, the genotypic relationship to other Chinese strains, and the relationship with human strains of HEV. Lots of evidences directly demonstrated that HEV infection is a food-borne zoonosis [27]–[29], especially identical HEV genomes have been isolated from patients and from meats they consumed. In the south of China, especially in Guangdong and Guizhou provinces (where Canton, Macao and Shenzhen fall within or near), the consumption of dog and cat meats were common, and this dietary habit has a long history in certain portions of the countryside. As stray dogs with a high level of anti-HEV antibody (OR, 2.40), eating dogs and cats should be given more attention. However, the presence of high levels of anti-HEV antibody in dogs and cats does not necessarily indicate virus transmission through contaminated meat. Further study should include specific RT-PCR for HEV detection in suitable samples.

In conclusion, this study presents a new result about seroprevalence of HEV of dogs and cats in several metropolitan area from China Dietary habits may be a potential risk for HEV transmission from humans to pets. However, there were some limitations to this study. Although we searched for evidence of HEV infection via one serological methods (DS-ELISA) and did not use a species-specific HEV EIA, it remains possible that true seropositivity with HEV infections among dogs and cats. Though this assay is not 100% sensitive, survey has been carried out to demonstrate it's species independence, high sensitive and agreement [15], [16]. To understand the epidemiology of HEV infection in humans and other animals, full-length HEV genome isolated from pets is required. The sample size was relatively small and may not be fully representative of China. Further investigation is required to confirm the conjecture, and an extensive public health surveillance system needs to be established and maintained in China.
